# Bimodal Nd-Doped LuVO_4_ Nanoprobes Functionalized with Polyacrilic Acid for X-Ray Computed Tomography and NIR Luminescent Imaging

**DOI:** 10.3390/nano10010149

**Published:** 2020-01-14

**Authors:** Nuria O Nuñez, Fernando Cussó, Eugenio Cantelar, Beatriz Martin-Gracia, Jesús M de la Fuente, Ariadna Corral, Marcin Balcerzyk, Manuel Ocaña

**Affiliations:** 1 Instituto de Ciencia de Materiales de Sevilla, CSIC-US, Américo Vespucio 49, Isla de la Cartuja, 41092 Sevilla, Spain; nurianu@icmse.csic.es; 2 Departamento de Física de Materiales, C-04, Universidad Autónoma de Madrid, 28049 Madrid, Spain; fernando.cusso@uam.es (F.C.); eugenio.cantelar@uam.es (E.C.); 3 Instituto de Ciencia de Materiales de Aragón, CSIC/UniZar and CIBER-BBN, Edificio I+D, Mariano Esquillor s/n, 50018 Zaragoza, Spain; beamar.91@hotmail.com (B.M.-G.); jmfuente@unizar.es (J.M.d.l.F.); 4 Centro Nacional de Aceleradores (CNA) (Universidad de Sevilla, Junta de Andalucía, CSIC), c/Thomas Alva Edison 7, Isla de la Cartuja, 41092 Sevilla, Spain; aricorral@gmail.com (A.C.); mbalcerzyk@us.es (M.B.); 5 Departamento de Fisiología Médica y Biofísica, Facultad de Medicina, Universidad de Sevilla, Calle San Fernando, 4, 41004 Sevilla, Spain

**Keywords:** nanoparticles, LuVO_4_, neodymium, polyacrilic acid, NIR luminescence, X-ray computed tomography

## Abstract

Uniform Nd^3+^-doped LuVO_4_ nanophosphors have been synthesized for the first time in literature by using a poliol-based method at 120 °C from Nd^3+^ and vanadate precursors. After optimizing the Nd doping level, these phosphors present intense luminescence in the near-infrared biological windows. The X-ray attenuation capacity of the optimum nanophosphor has been found to be higher than that of a commercial X-ray computed tomography contrast agent. After surface coating with polyacrylic acid, such nanoparticles present high colloidal stability in physiological pH medium and high cell viability. Because of these properties, the developed Nd^3+^-doped LuVO_4_ nanoparticles have potential applications as a bimodal probe for NIR luminescent bioimaging and X-ray computed tomography.

## 1. Introduction

Among the most interesting techniques for medical diagnosis are luminescent imaging (LI), magnetic resonance imaging (MRI), and X-ray computed tomography (CT) [1]. In both cases, the use of external probes as contrast agents is usually required. Several compounds have been assayed for such a purpose, among which rare earth vanadate (REVO_4_)-based nanophosphors composed by a REVO_4_ matrix in which different luminescent lanthanide (Ln) cations are introduced as dopants, have been recently the focus of much attention. In particular, Ln-doped REVO_4_ (RE = Y, La, Gd; Ln = Eu, Tb, Dy, Sm, Nd, Yb), have been proposed as excellent probes for luminescent “in vitro” bioimaging and biosensing [2,3,4,5,6,7,8,9,10], owing to their intense luminescence on excitation by UV light through an energy transfer process from the vanadate matrix to the Ln^3+^ cations [11]. However, the need for UV excitation is a drawback for in vivo bioapplications because of the potential damage that such radiation may cause to living organisms. It has been shown that for such applications, the use of doping Ln^3+^ cations whose luminescent properties are located within the biological windows in the near-infrared (NIR) region would be more appropriate, as it is the case of Nd^3+^ [12].

REVO_4_-based multifunctional probes useful for more than one imaging technique have been also reported. For example, Ln-doped GdVO_4_ (Ln = Eu, Dy) nanoparticles have been shown to be promising candidates as both luminescent and MRI probes due to the magnetic properties of the Gd^3+^ cations [2,3,4,5]. In addition, because the atomic number of Ln is high, the X-ray attenuation capability of these elements is also high [13], and consequently, Ln-based nanoparticles can be also used as contrast agents for CT. For that reason, Lu^3+^-based compounds are the most advantageous probes for this application. However, in the case of LnVO_4_ compounds, this additional functionality has been only studied for the of case Ln-doped Y(Gd)VO_4_ (Ln = Eu, Dy, Sm) nanoparticles [14]. It should be also noticed that no reports on the development of luminescent Nd:LuVO_4_-based bioprobes could be found in the literature either. The lack of antecedents regarding to the potential use of this material for bioimaging purposes might be due to the scarce availability of synthesis methods yielding Ln-doped LuVO_4_ particles with appropriated characteristics for such uses, namely, uniformity in size and shape, particle size lower than 100 nm, low cytotoxicity, and high colloidal stability in physiological media [15,16].

In this paper, we report on a procedure for the synthesis, for the first time in the literature, of uniform Nd^3+^-doped LuVO_4_ nanophosphors with nearly equiaxial shape by homogeneous precipitation at 120 °C from ethylene glycol (EG)/water solutions containing Ln^3+^ nitrates and sodium orthovanadate. The effects of the amount of Nd^3+^ incorporated into the LuVO_4_ matrix on the luminescence of the obtained nanoparticles have been studied aiming to establish the optimum Nd^3+^ content. The X-ray attenuation capacity of the optimized phosphor has been also evaluated in comparison with that of Iohexol, one of the CT contrast agents most used in clinics. To achieve the colloidal stability criteria in physiological pH conditions, the optimum sample has been surface coated with polyacrylic acid (PAA) molecules and the cell viability of such functionalized samples has been also analyzed. This study demonstrates that the developed Nd^3+^:LuVO_4_ nanophosphors are promising candidates for their use as a bimodal probe for in vivo luminescent bioimaging and X-ray computed tomography.

## 2. Materials and Methods

### 2.1. Materials

Lutetium (III) nitrate hydrate (Lu(NO_3_)_3_·xH_2_O, Sigma-Aldrich, 99.9%, St. Louis, MO, USA), neodymium (III) nitrate hexahydrate (Nd(NO_3_)_3_·6H_2_O, Sigma-Aldrich, 99.9%, St. Louis, MO, USA), sodium orthovanadate (Na_3_VO_4_, Sigma-Aldrich, 99.9%, St. Louis, MO, USA), ethylene glycol (EG, Sigma-Aldrich, 99.9%, St. Louis, MO, USA), polyacrylic acid (PAA, average Mw~1800, Sigma-Aldrich, St. Louis, MO, USA), 2-morpholinoethanesulfonic acid (MES, Sigma-Aldrich, 99%, St. Louis, MO, USA), and Iohexol (Sigma-Aldrich, analytical standard, ≥95%, St. Louis, MO, USA) were used as received.

### 2.2. Synthesis of LuVO_4_ Nanoparticles

Nd-doped LuVO_4_ nanoparticles having different Nd contents (Nd/(Nd + Lu) mol ratio = 0, 0.2, 0.5, 1.0, 1.5, 2.0) were synthesized by homogeneous precipitation according to a procedure previously developed by us for the preparation of GdVO_4_ nanoparticles [2], which was adapted by introducing the needed modifications in order to produce nanoparticles of the desired compound with narrow size distribution. Specifically, for a given doping level, the required amounts of the lanthanide precursors (Lu(NO_3_)_3_ and Nd(NO_3_)_3_, total Ln concentration = 0.04 moldm^−3^), and Na_3_VO_4_ (0.04 mol dm^−3^) were separately dissolved in Milli-Q water (0.75 cm^3^) under magnetic stirring. EG (1.75 cm^3^) was then added to each of these solutions. After homogenization, they were mixed under vigorous stirring. The resulting mixture (total volume = 5 cm^3^) introduced in a test tube, which was tightly closed, was aged for 20 h in an oven at constant temperature (120 °C). The obtained dispersions were allowed to cool to room temperature. The precipitates were purified by centrifugation and redispersion, twice in ethanol and once in distilled water. The as-prepared particles were stored dispersed in Milli-Q water and dried at room temperature when required.

### 2.3. PAA Functionalization of LuVO_4_ Nanoparticles

PAA (2 mg cm^−3^) was dissolved in Milli-Q water containing 0.5 mg cm^−3^ of Nd:LuVO_4_ nanoparticles. After adjusting the pH of this dispersion to 10 by adding a few droplets of a NaOH solution, it was maintained for 1 h at −25 °C under magnetic stirring. Afterward, the supernatants were removed by centrifugation and the surface-modified nanoparticles were washed several times with Milli-Q water and finally dispersed in Milli-Q water.

### 2.4. Characterization

Transmission electron micrographs (TEM) were taken in a Philips 200CM microscope (ThermoFisher Scientific, Hillsboro, OR, USA). Particle size distributions were estimated from the TEM pictures by counting hundreds of particles.

X-ray diffraction (XRD) patterns were recorded in a Panalytical X′Pert Pro diffractometer (Malvern Panalytical, Cambridge, UK) equipped with an X-Celerator detector. Unit cell constants were calculated by Rietveld refinement of the XRD patterns (registered with a 0.02° 2θ step and an accumulation time of 1000 s) using the X′Pert High Score Plus software.

The infrared spectra (FTIR) of the samples diluted in KBr were measured in a JASCO FT/IR-6200 apparatus (Jasco Europe SRL, Cremella, Italy).

Thermogravimetric analyses (TGA) were conducted in air atmosphere employing a heating rate of 10 °C min^−1^ in a Q600 TA instrument (TA instrument, New Castle, DE, USA).

Excitation and emission spectra of the samples in powdered form were obtained using a tunable Ti:Sapphire laser as excitation source. To obtain comparative data, we employed the same amount of sample, which was placed in a hole (3 mm diameter) practiced in an aluminum foil, which was sandwiched between two microscope slides. Lifetime measurements were carried out by exciting the samples at 532 nm with the second harmonic of a pulsed Nd:YAG laser (pulse width, 10 ns and repetition rate, 10 Hz) and recording the luminescence decay at λ_exc_ = 1344 nm (^4^F_3/2_ → ^4^I_13/2_ transition) using a Tektronix DPO4104B-L digital oscilloscope.

CT measurements were acquired with a NanoSPECT/CT^®^ apparatus (Mediso, Budapest, Hungary). For such a purpose, different amounts of the sample were dispersed in Milli-Q and the dispersion was homogenized in a vortex device for 2 min before measurements. Afterward, 200 μL of each dispersion was introduced in a multiwell microplate, along with a Milli-Q water aliquot used for calibration. The acquisition parameters were: 106 mA current for a voltage of 65 kV, exposure time per projection of 1500 ms, 360 projections per rotation, and total acquisition time of 18 min. The image length was 6 cm, with a pitch of 1. The image was reconstructed with Vivoquant image processing software (Invicro, London, UK), with the exact cone-beam filtered back-projection algorithm and the Shepp Logan 98% filter. The resulting image pixel size was uniform in three dimensions at 0.2 mm. Images were analyzed with PMOD 3.8 software (PMOD Technologies LLC, Zürich, Switzerland). Spherical volumes of interest (VOIs) of a 2 mm radius were made within each sample to calculate the X-ray attenuation (in Hounsfield Units, HU) for each concentration. Average values of Milli-Q water and dispersions were used to calculate HU values in the images, with attenuation being 0 HU for water and −1000 HU for air.

The colloidal stability of the dispersions containing the nanophosphors (0.1 mg cm^−3^) in 50 mM MES solution at pH = 6.5 was evaluated by measuring the hydrodynamic diameter (d_h_) by dynamic light scattering (DLS) using a Zetasizer NanoZS90, Malvern instrument.

To analyze cytotoxicity, the 3-(4,5-dimethylthiazol-2-yl)-2,5-diphenyltetrazolium bromide (MTT) colorimetric assay [17] and Vero cells (kidney epithelial cells from African green monkey) were used. The cells, purchased from the American Type Culture Collection (ATCC: CCL-81), were cultured in a 5% CO_2_ atmosphere in Dulbecco’s modified Eagle’s medium (DMEM) supplemented with 10% fetal bovine serum (FBS), 2 mM glutamine, and 100 U cm^−3^ penicillin/streptomycin at 37 °C.

For the MTT analysis, 5000 cells were first seeded in each well of 96-well plates and grown for 24 h. Then, the medium was substituted with fresh medium containing variable amounts of nanoparticles. After further cultivation for 24 h, 20 µL of MTT dye solution (5 mg cm^−3^ in PBS) was added to each well. After 3 h of incubation at 37 °C and 5% CO_2_, the medium was removed, the cells were washed with fresh medium, the plate was centrifuged at 2500 rpm for 30 min, the supernatant was discarded, and formazan crystals were dissolved in 100 µL of DMSO. The absorbance of each well was read on a microplate reader (Multiskan GO, Thermo Scientific, Waltham, MA, USA) at 570 nm. The relative cell viability (%) related to control wells containing cells without nanoparticles was calculated as [A]test/[A]control × 100.

## 3. Results

### 3.1. Samples Preparation and Characterization

**Figure 1:**
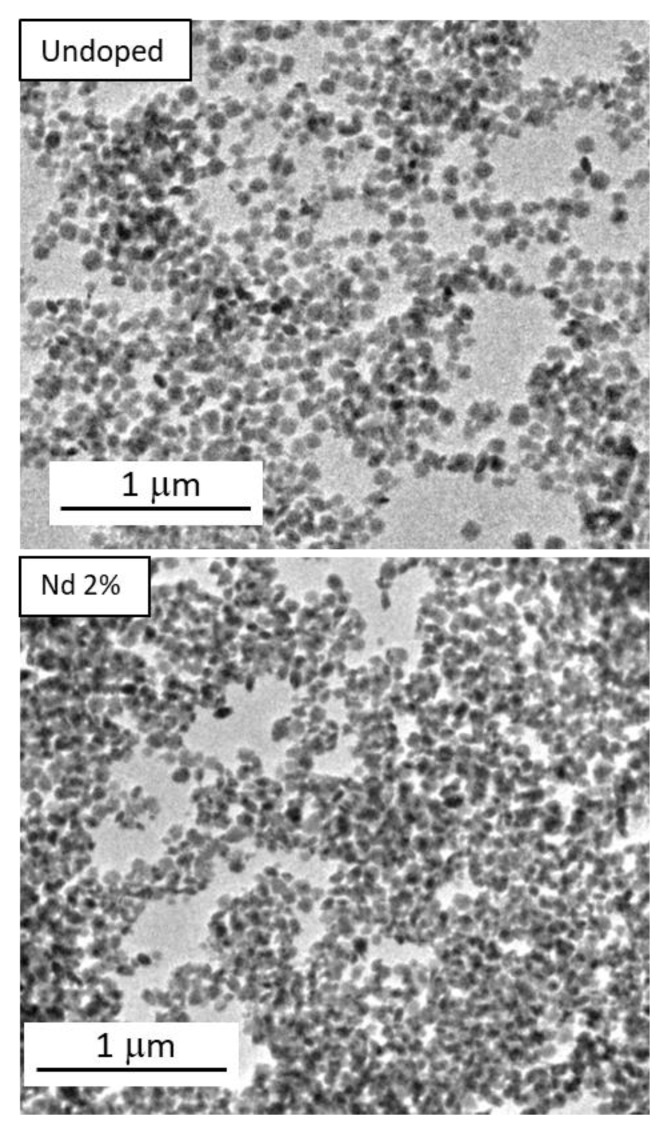
TEM images of the particles synthesized by precipitating solutions in EG/H_2_O (3.5/1.5) containing: (**top**) Na_3_VO_4_ (0.04 mol dm^−3^) and Lu(NO_3_)_3_ = 0.04 mol dm^−3^; (**down**) under the same conditions but in the presence of 2% Nd (Nd/(Nd + Lu) mol ratio). In both cases, the aging temperature was set at 120 °C and aging time was 20 h.

The aging at 120 °C for 20 h of Ln nitrate (Lu(NO_3_)_3_ + Nd(NO_3_)_3_ = 0.04 mol dm^−3^ and Nd/(Nd + Lu) mol ratio from 0.2% to 2%) and sodium orthovanadate (0.04 mol dm^−3^) solutions in EG/water (3.5/1.5 by volume) yielded almost equiaxed nanoparticles as those illustrated in Figure 1, in which the undoped sample and that with the highest doping level are shown.

The particle size, as measured from the TEM micrographs, was found to be ~70 nm for all samples, irrespective of the doping level (Table 1).

**Figure 2:**
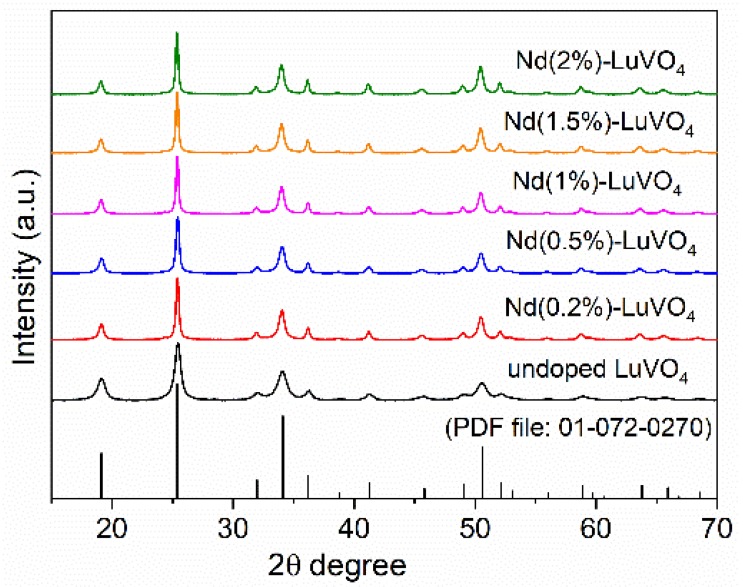
X-ray diffraction patterns for the nanoparticles obtained in the absence (undoped) and the presence of different amounts of Nd^3+^, and PDF of tetragonal LuVO_4_.

According to XRD, the obtained nanoparticles consisted of tetragonal LuVO_4_ (PDF 1-72-270, ICDD 2016), irrespective of the Nd content (Figure 2). The successful incorporation of Nd^3+^ into the LuVO_4_ lattice was assessed by the evaluation of the unit cell constants of the samples. As observed in Table 1, the increase of the Nd content gave rise to a progressive unit cell expansion, which manifests that the Lu^3+^ cations have been substituted by Nd^3+^ in the LuVO_4_ structure since the ionic radius in of Nd^3+^ (1.109 Å) is greater than that of Lu^3+^ (0.977 Å), both in eight-fold coordination [18].

### 3.2. NIR Luminescence

**Figure 3:**
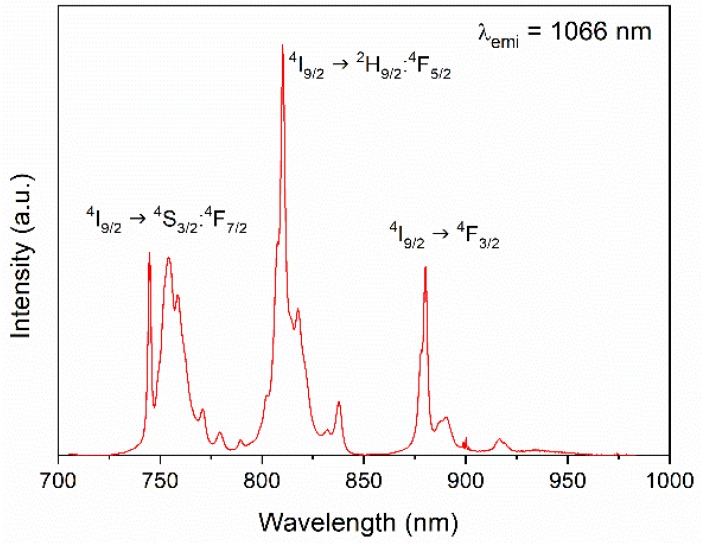
Excitation spectra of the Nd (0.2%):LuVO_4_ nanoparticles.

The number, position, and relative intensity of the excitation bands registered for the emission appearing at 1066 nm for the Nd:LuVO_4_ nanoparticles were similar, irrespective of the Nd content. As an illustrative example, the spectrum of the sample doped with a 0.2% molar of Nd^3+^ is shown in Figure 3. As expected, this spectrum shows three groups of bands in the 730–790 nm, 790–850 nm, and 870–930 nm regions, associated to the ^4^I_9/2_ → ^4^S_3/2_:^4^F_7/2_, ^4^I_9/2_ → ^2^H_9/2_:^4^F_5/2_, and ^4^I_9/2_ → ^4^F_3/2_ f-f electronic transitions of the Nd^3+^ ions, respectively [7].

**Figure 4:**
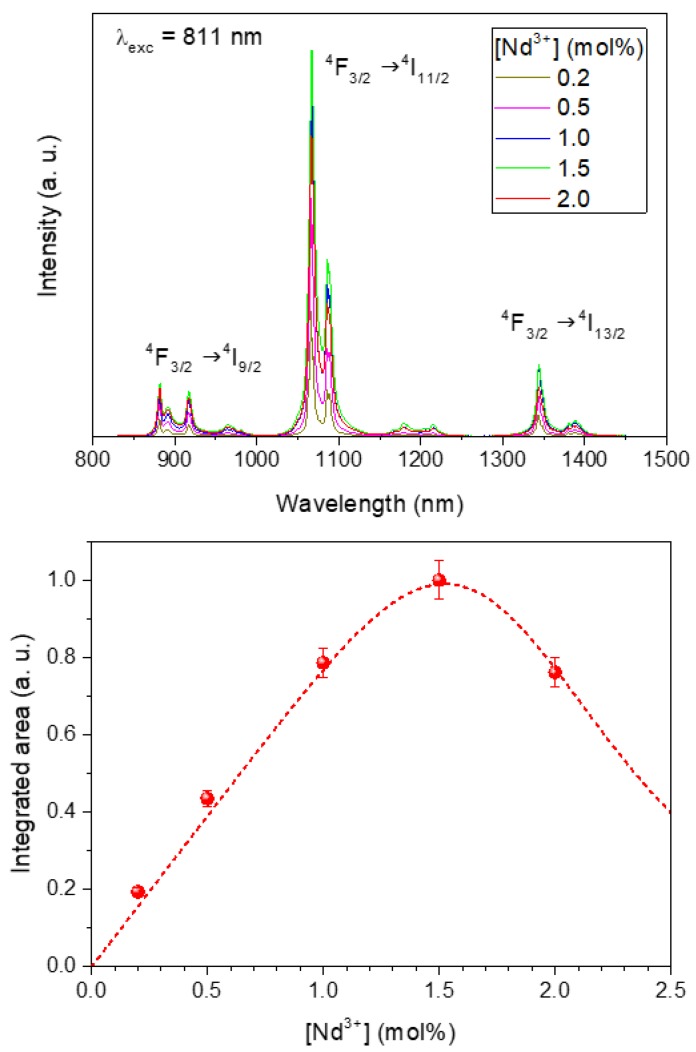
(**Top**) Emission spectra of the Nd:LuVO_4_ nanoparticles with different Nd^3+^ content. (**Down**) Integrated intensity of the emissions vs. the Nd^3+^ doping level. Line is a guide for the eye. Error bars have been included.

The emission spectra of the LuVO_4_ nanoparticles containing different amounts of Nd^3+^ obtained upon excitation through the most intense excitation band (λ_ex_ = 810 nm) are presented in Figure 4 (top). The emission bands characteristic of the ^4^F_3/2_ → ^4^I_J_ (J = 9/2, 11/2 and 13/2) electronic transitions of Nd^3+^ were detected in all cases [7], whose intensity varies with the doping level. Thus, when plotting the integrated area of the emissions vs. the Nd content (Figure 4 (down)), it was clearly observed that the rise of the Nd^3+^ doping level up to 1.5% produced a progressive increase of the emission intensity, which must be attributed the increase of the number of emission centers. However, above such doping levels (2%), the emission intensity decreased, suggesting the presence of concentration quenching. This known effect was further confirmed by determining the luminescence lifetime of the samples.

**Figure 5:**
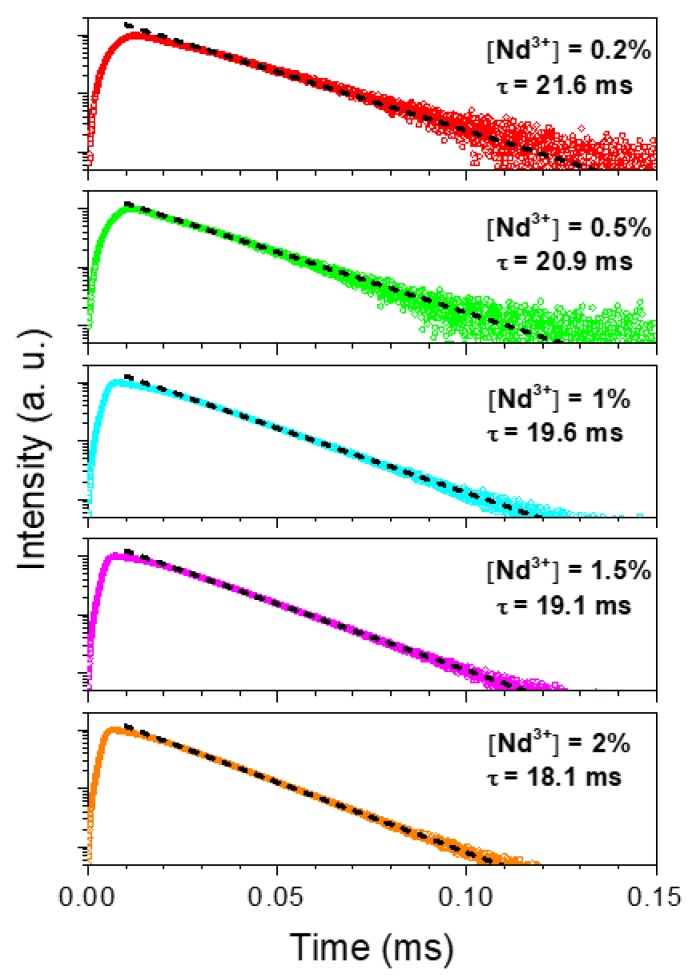
Temporal decays for the ^4^F_3/2_ → 4I13_/2_ transition (1344 nm) of the Nd:LuVO_4_ nanoparticles with different Nd^3+^ content in a semi-logarithmic plot. The decay time values correspond to the least squares fitting to Equation (1).

The decay curves corresponding to the ^4^F_3/2_ → ^4^I_13/2_ Nd^3+^ transition (1344 nm) of the Nd:LuVO_4_ samples are presented in Figure 5 in a semi-logarithmic plot. As it can be observed, after an initial rise, the luminescence temporal evolution decays following a single exponential dependence:

*I*(*t*) = *I*_0_ exp(−*t*/*τ*) (1)

where *I*(*t*) is the luminescence intensity, *I*_0_ is the initial intensity, *t* is the time after excitation, and *τ* is the decay time. Dashed lines in Figure 5 correspond to least squares fitting.

**Figure 6:**
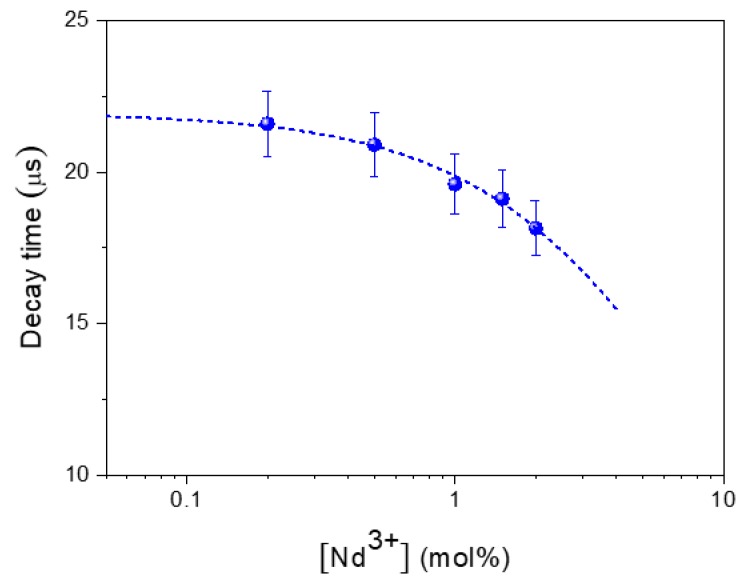
Concentration dependence of the experimental decay times. Dashed line represents the least squares fitting to Equation (1). Error bars have been included.

The corresponding decay time values obtained from this fitting are also included in Figure 5, and their evolution with Nd^3+^ concentration is plotted in Figure 6. As observed, the decay time decreases monotonously with Nd^3+^ content, indicating the occurrence of concentration quenching processes, which become more evident at doping levels ≥1% Nd. This implies a reduction in the luminescence efficiency that counterbalances the rise in the number of emitting centers as concentration increases. According to the results obtained under CW excitation (Figure 5), the best compromise seems to happen for the sample having a 1.5% Nd, which can be considered, from a practical point of view, as the optimum nanophosphor for bioimaging owing to its highest emission intensity.

### 3.3. X-Ray Attenuation

The X-ray attenuation properties of the optimum nanophosphor (Nd (1.5%):LuVO_4_) and those of Iohexol, which is one of the most commonly used CT contrast agents in clinics, were measured in aqueous suspensions.

**Figure 7:**
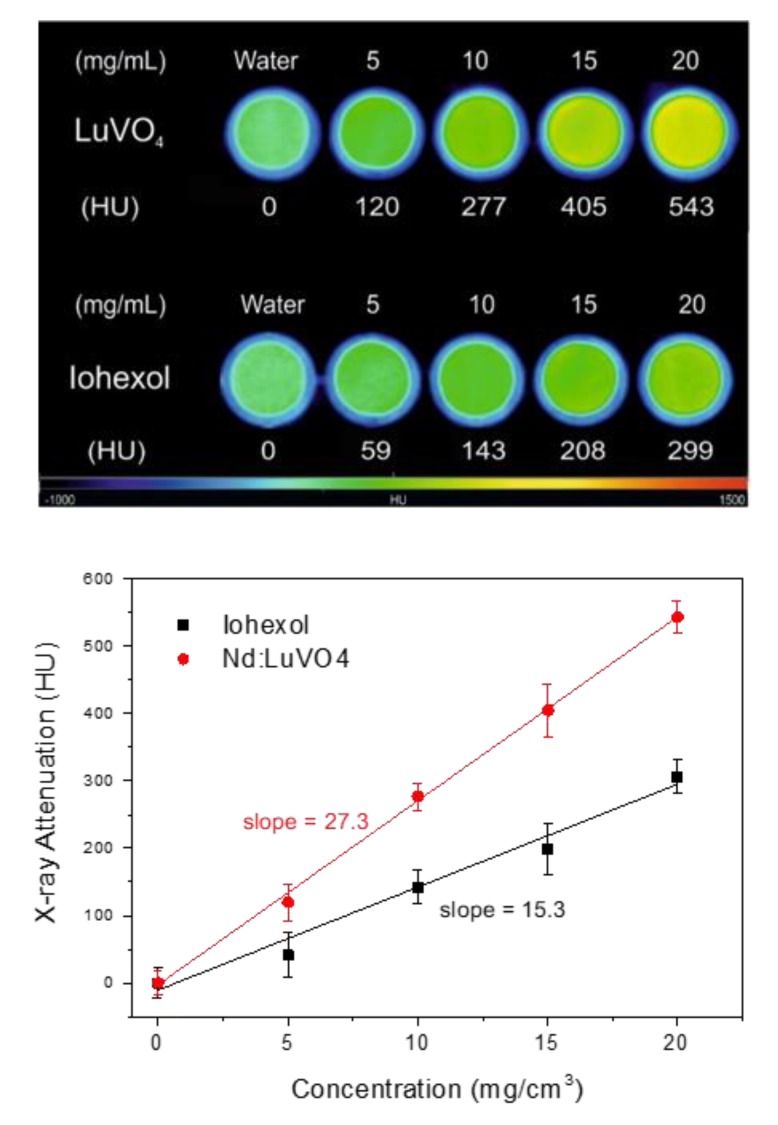
X-ray attenuation phantom images (**top**) and Hounsfield units (HU) values (**bottom**) obtained for the Nd (1.5%):LuVO_4_ nanoparticles and for Iohexol at different concentrations in water.

The obtained X-ray attenuation phantom images are shown in Figure 7 (top), where it can be clearly observed that the contrast produced by increasing the nanophosphor’s concentration in the suspensions is higher than that corresponding to Iohexol, suggesting better performance of our nanoparticles as a CT contrast agent. This finding was further corroborated by the X-ray attenuation values, in Hounsfield units (HU), which were estimated from the phantom images, and plotted in Figure 7 (bottom) as a function of the nanophosphor’s concentration. This figure reveals an increase of the HU values by increasing the concentration of the suspensions following a straight line whose slope is higher for our system (27.3 HU/mg cm^−3^) than for Iohexol (15.3 HU/mg cm^−3^). This behavior is not surprising given the expected increase of the X-ray attenuation coefficient when the average atomic number of the elements constituting the probe increases [13]. The excellent X-ray attenuation properties of our nanophosphors indicate that they can be used not only as a probe for NIR imaging but also as a CT contrast agent.

### 3.4. Colloidal Stability and PAA Surface Modification

The colloidal stability of our optimum bifunctional probe (Nd (1.5%):LuVO_4_) was evaluated through DLS measurements conducted in freshly prepared suspensions containing our nanoparticles in MES buffer at physiological pH (MES 50 mM at pH = 6.5).

**Figure 8:**
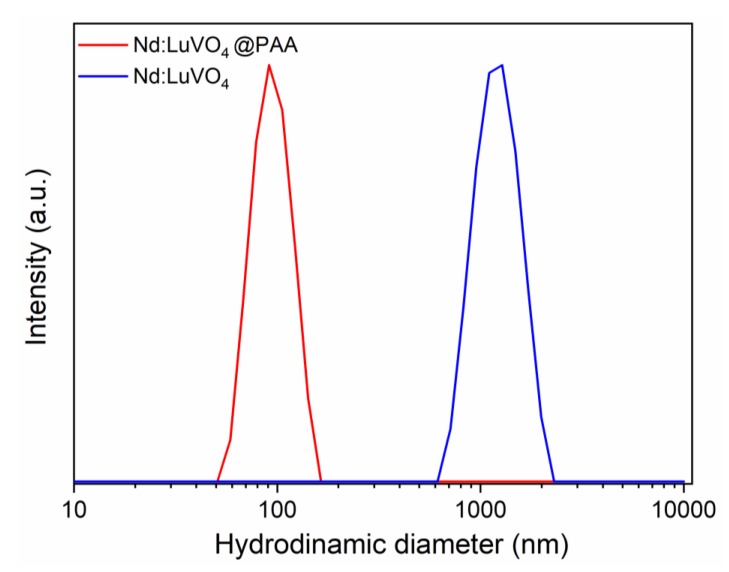
DLS curves obtained for the Nd (1.5%):LuVO_4_ nanoparticles before and after surface functionalization with polyacrylic acid (PAA) and dispersed in 50 mM MES solution at pH = 6.5. The hydrodynamic diameter (d_h_) is also included.

**Figure 9:**
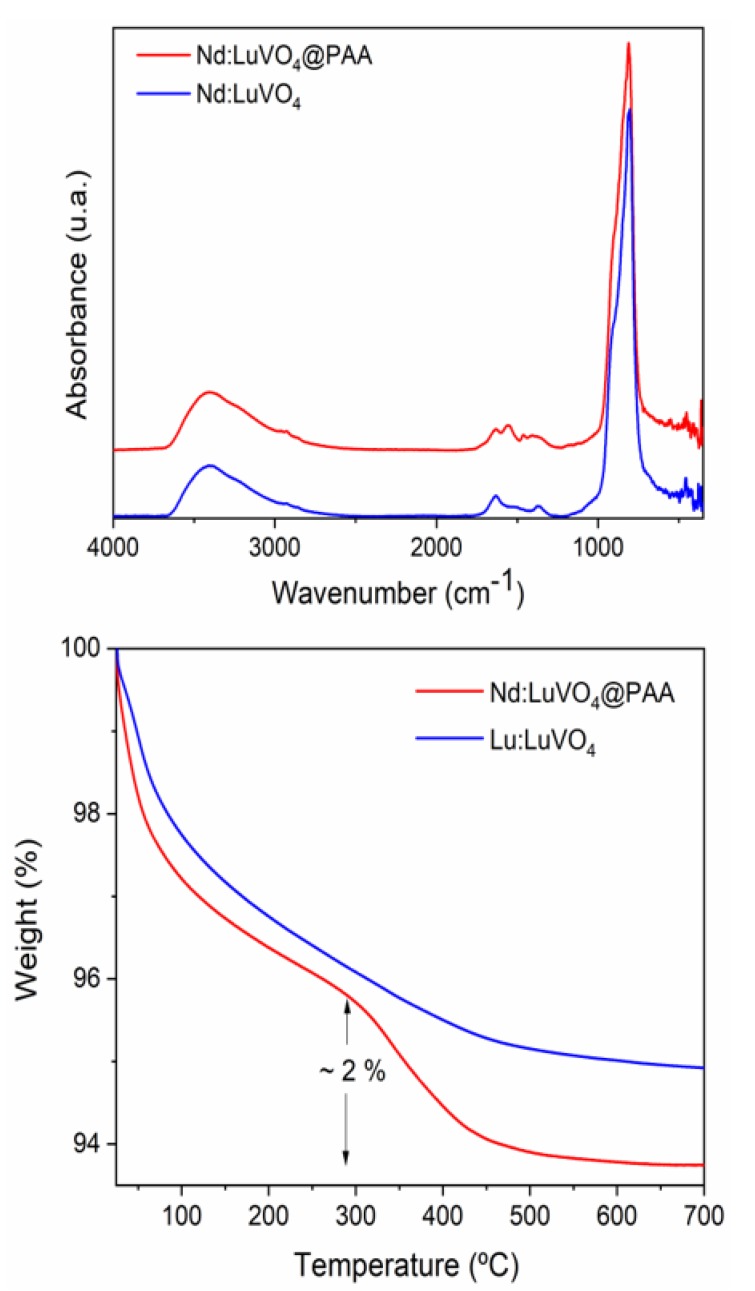
FTIR spectra (**top**) and TGA curves (**bottom**) obtained for the Nd (1.5%):LuVO_4_ nanoparticles before and after surface functionalization with PAA.

It was found that the hydrodynamic diameter (d_h_) obtained from the DLS curve (Figure 8) was much higher (1280 nm) than the mean particle size resulting from the analysis of the TEM pictures (77 nm) indicating that this sample does not meet the stability criterion established for the use for in vivo applications. To overcome this limitation, a surface modification approach, consisting of a coating process of the nanoparticles with PAA polymer, was undertaken. After such a process, a surface charge reversal was detected. Thus, the zeta potential value changed from +25 mV to −45 mV after functionalization, which suggests the presence of PAA molecules with ionized carboxylate groups on the particle surface. These species could be identified in the FTIR spectra of the functionalized sample. Thus, the spectrum of the pristine sample (Figure 9, top) only displayed the absorption features associated with the vanadate vibrational modes (<1000 cm^−1^) [7], adsorbed water (3400 and 1625 cm^−1^), and a very weak band at 1370 cm^−1^ probably due to residual EG molecules [19]. However, additional weaker features in the 1600–1400 cm^−1^ region were detected in the spectrum of the functionalized nanoparticles, which can be attributed to the asymmetric (1545 cm^−1^) and symmetric (1400 cm^−1^) stretching vibrations of the carboxylate groups of the PAA molecules [7]. These results confirm that our functionalization procedure was successful.

The amount of PAA polymer deposited on the nanoparticle surface could be quantified according to the TGA curves presented in Figure 9 (bottom). As observed, in the curve corresponding to the original sample a first weight loss (~4%) was detected in the 25–300 °C range due to the removal of adsorbed water. The smaller loss (1.5%) that occurred at higher temperatures (300–700 °C) might be a consequence of the decomposition of the residual EG molecules. The functionalized sample presented an additional weight loss (2%) between 300 and 500 °C, which must be due to the decomposition of PAA entities.

The PAA coating gave rise to an improvement in the colloidal stability of the nanophosphor. Thus, the value of d_h_ obtained in MES media after functionalization (Figure 8) was closer (90 nm) to the mean particle size (77 nm), clearly indicating the absence of significant aggregation, as required for the intended biomedical application of the developed probe.

### 3.5. Biocompatibility

**Figure 10:**
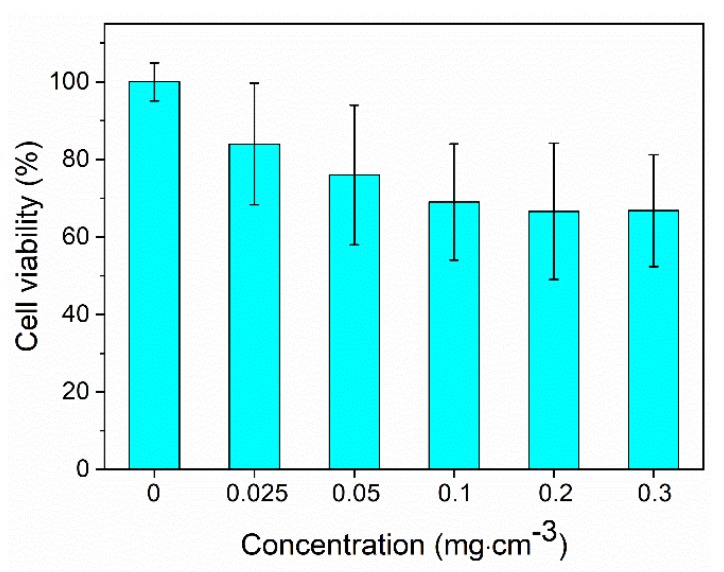
Cell viability of the Nd (1.5%):LuVO_4_ nanoparticles functionalized with PAA.

Cell viability analyses were performed for the optimum nanophosphor using the MTT assay [17] with Vero cells. The metabolic activity of the cells measured for different nanoparticles concentrations after 24 h culture is shown in Figure 10, in which it can be observed that cell survival remained above 65% for nanoparticle contents in the suspensions up to 0.3 mg cm^−3^.

## 4. Conclusions

The aging at 120 °C for 20 h of solutions containing precise amounts of Lu(NO_3_)_3_, Nd(NO_3_)_3_, (Lu(NO_3_)_3_ + Nd(NO_3_)_3_ = 0.04 mol dm^−3^ and variable Nd/(Nd + Lu mol ratio), and sodium orthovanadate (0.04 mol dm^−3^) in EG/H_2_O medium (3.5/1.5 ratio, by volume) yields Nd^3+^-doped LuVO_4_ nanoparticles with almost equiaxed shape and narrow size distribution (~70 nm). These particles crystallize into the tetragonal phase and show polycrystalline character. Irrespective of the Nd content (from 0.2% to 2%), NIR emissions in the 850–1400 nm region were detected for all samples upon excitation at 811 nm. The most intense emissions corresponded to the particles doped with 1.5 mol% Nd^3+^, for which they were considered as the optimum nanophosphors for bioimaging. The X-ray attenuation properties of this nanophosphor were higher than that of Iohexol, a commercial X-ray computed tomography contrast agent. After surface coating with polyacrylic acid molecules, this probe showed high colloidal stability in aqueous suspensions at physiological pH and low cytotoxicity. Because of these properties, the Nd-doped LuVO_4_ nanoparticles functionalized with polyacrylic acid can be considered as promising candidates for their use as bimodal probes for NIR luminescent bioimaging and X-ray computed tomography.

## Figures and Tables

**Table 1:** Mean size, unit cell parameters, cell volume, and crystallite size of the particles obtained by aging at 120 °C for 20 h. EG/H_2_O solutions containing Lu(NO_3_)_3_, Nd(NO_3_)_3_, (Nd(NO_3_)_3_ + Lu(NO_3_)_3_ = 0.04 mol dm^−3^, and variable Nd/(Nd + Lu mol ratio) and sodium orthovanadate (0.04 mol dm^−3^) in EG/H_2_O medium (ratio by volume = 3.5/1.5). Standard deviations for particle size and unit cell parameters errors are included in parentheses.

Nd/(Nd + Lu)	Mean Size	a = b	c	Cell Volume
(% Nominal)	(nm)	(Å)	(Å)	(Å^3^)
**0**	60 (14)	7.0330(9)	6.2180(1)	307.58
**0.2**	73 (11)	7.0305(7)	6.2274(7)	307.80
**0.5**	70 (12)	7.0332(7)	6.2280(7)	308.07
**1.0**	75 (9)	7.0341(6)	6.2296(6)	308.20
**1.5**	77 (13)	7.0352(5)	6.2294(5)	308.31
**2.0**	75 (10)	7.0367(6)	6.2321(6)	308.58
